# [Retracted] Long non‑coding RNA urothelial cancer associated 1 can regulate the migration and invasion of colorectal cancer cells (SW480) via myocardin‑related transcription factor‑A

**DOI:** 10.3892/ol.2024.14760

**Published:** 2024-10-21

**Authors:** Long Zhang, Chengcheng Zheng, Zhen Sun, Huaqing Wang, Fengwei Wang

Oncol Lett 18: 4185–4193, 2019; DOI: 10.3892/ol.2019.10737

Following the publication of this paper, it was drawn to the Editor's attention by a concerned reader that certain of the Transwell invasion assay data shown in Fig. 1C on p. 4187 and Fig. 4E on p. 4190 were strikingly similar to data that had either already been published, or were already under consideration for publication, in a pair of articles written by different authors at different research institutes; in addition, a pair of western blots had been duplicated internally within Figs. 2B and 5B. In a separate communication, the authors contacted the Editorial Office to request that this article be retracted. Following on from the authors’ request, the Editor of *Oncology Letters* has agreed that this paper should be retracted from the Journal. The Editor would like to apologize to the readership for any inconvenience caused.

